# The Deceptive Nature of Pneumatosis Intestinalis: From Spontaneous Resolution to Bowel Ischemia

**DOI:** 10.31486/toj.25.0075

**Published:** 2026

**Authors:** Carolina Baz, Ian Bussey, Jennifer Wormuth

**Affiliations:** Department of Surgery, Luminis Health Anne Arundel Medical Center, Annapolis, MD

**Keywords:** *Bowel*, *conservative treatment*, *ischemia*, *physiopathology*, *pneumatosis cystoides intestinalis*, *surgery*

## Abstract

**Background:**

Pneumatosis intestinalis, a rare condition characterized by the presence of gas within the bowel wall, has an estimated incidence of 0.03% in the general population. Although the exact etiology of the condition remains uncertain, pneumatosis intestinalis is broadly classified as primary (idiopathic) or secondary (associated with an underlying condition), and the secondary form accounts for approximately 85% of cases. Secondary pneumatosis intestinalis has been associated with up to 60 potential causes and presents with a wide range of clinical manifestations, making thorough physical examination and imaging essential for appropriate management. Diagnosis is typically based on characteristic computed tomography (CT) findings.

**Case Report:**

An 89-year-old female presented with shortness of breath, abdominal pain, nausea, vomiting, and diarrhea. CT imaging revealed extensive pneumatosis intestinalis in the small intestine. Despite the patient's stable hemodynamics and a mostly unremarkable physical examination, focal abdominal tenderness raised concern for bowel ischemia. The patient underwent an exploratory laparotomy that revealed patchy areas of pneumatosis intestinalis in an otherwise normal-appearing small bowel, with no evidence of ischemia or necrosis.

**Conclusion:**

Because of the deceptive nature of pneumatosis intestinalis, the mere presence of the condition is not enough to justify surgery. Cases with benign causes can often be managed conservatively and resolve spontaneously, whereas concerning findings that may indicate a surgically treatable cause may require urgent intervention to reduce mortality.

## INTRODUCTION

Pneumatosis intestinalis is an uncommon condition characterized by the presence of gas within the intestinal wall, typically affecting the mucosa and submucosa of the small and large intestines.^1,2^ The gas formation is categorized as bubble-like (cysts in the intestinal wall) or band-like (continuous lines),^3^ with the cysts located near blood vessels primarily on the antimesenteric aspect of the bowel.^4^ While the band-like pattern is potentially indicative of bowel ischemia or portal venous gas, the bubble-like pattern is often harmless.^3^

Defined as a radiographic diagnosis by Lerner and Gazin in 1946,^5^ pneumatosis intestinalis can occur at any age, depending on the underlying cause. Up to 60 causes—both benign and life-threatening etiologies—are associated with pneumatosis intestinalis,^6^ which is classified as primary or secondary. The primary or idiopathic type (not associated with any other coexisting diseases) accounts for 15% of cases, while the secondary type, associated with a wide variety of underlying conditions, comprises the remaining 85% and includes life-threatening pathologies.^7,8^ Mesenteric ischemia, bowel obstruction, and bowel necrosis are common life-threatening causes. Pneumatosis intestinalis may also result from nonischemic and nonobstructive conditions, most of which are not linked to adverse outcomes.^9^

Patients with pneumatosis intestinalis may be asymptomatic or exhibit nonspecific symptoms such as abdominal pain, distention, and vomiting.^10,11^ Given the potential for an ambiguous presentation, which can easily lead to a misdiagnosis, a thorough evaluation of the patient is essential, including a comprehensive history, identification of specific signs, and review of laboratory results.^9,12,13^ Delays in surgical intervention can increase mortality.^9^ Computed tomography (CT) scans are the gold standard imaging modality because they demonstrate greater sensitivity than plain radiography in differentiating between benign and concerning pneumatosis intestinalis.^1,14^

The priority in management is to determine whether the underlying pathology is benign or life-threatening. If a benign cause is suspected, conservative treatments are effective in 90% of cases.^15^ Surgical management is reserved for instances in which conservative treatment fails, etiology is unclear, or a life-threatening condition is identified.^15,16^

## CASE REPORT

**Figure 1.**
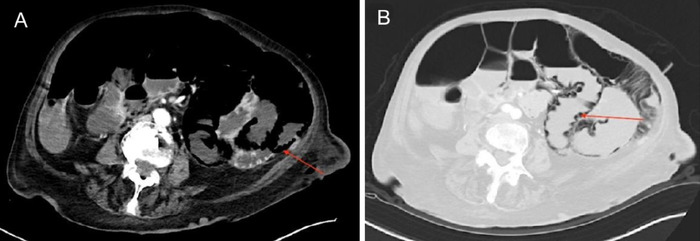
Computed tomography with intravenous contrast (A, B) axial views of the abdomen and pelvis show diffuse pneumatosis intestinalis (arrows) in the small bowel.

**Figure 2.**
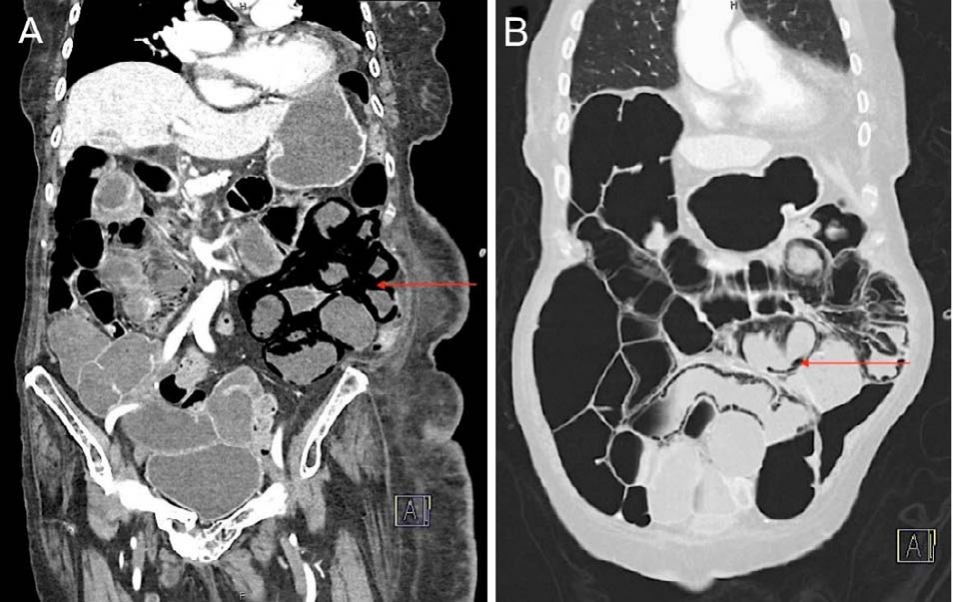
Computed tomography with intravenous contrast (A, B) coronal views of the abdomen and pelvis show diffuse pneumatosis intestinalis (arrows) in the small bowel.

An 89-year-old female with a history of hypertension, gastroesophageal reflux disease, asthma, and duodenal ulcer perforation status post Graham patch repair was brought to the emergency department (ED) from her assisted living facility because plain radiographs showed left-sided thoracic infiltrate and possible pneumoperitoneum. Beginning 1 day prior, the patient complained of abdominal pain, nausea, vomiting, and nonbloody diarrhea. In the ED, she appeared mildly dyspneic, her abdomen was soft without peritoneal signs, and she had tenderness in the left lower quadrant. Initially, the patient was reportedly hypotensive, but she showed a sustained response to fluid administration. Complete blood workup revealed leukocytosis of 16.23 × 10^3^/μL (reference range, 4.30-10.50 × 10^3^/μL) and hyponatremia of 127 mmol/L (reference range, 135-146 mmol/L); other laboratory findings were unremarkable, including a normal lactic acid level. Chest radiograph exhibited elevation of the left hemidiaphragm with loss of left basilar lung volume but no evidence of pneumoperitoneum. CT scan of the abdomen and pelvis with intravenous contrast showed distended loops of small bowel with pneumatosis intestinalis in the left abdomen but no evidence of pneumoperitoneum, bowel wall thickening, or portal venous gas (Figures 1 and 2).

Given the patient's overall stability and reassuring physical examination, a repeat CT scan with oral contrast was performed, which confirmed the previous findings and showed no evidence of mechanical bowel obstruction or contrast extravasation. However, because of the patient's initial hypotension, leukocytosis, hyponatremia, and persistent abdominal tenderness localized in the area where imaging showed the segment of small bowel with pneumatosis intestinalis, surgical exploration was recommended to rule out possible bowel ischemia.

**Figure 3.**
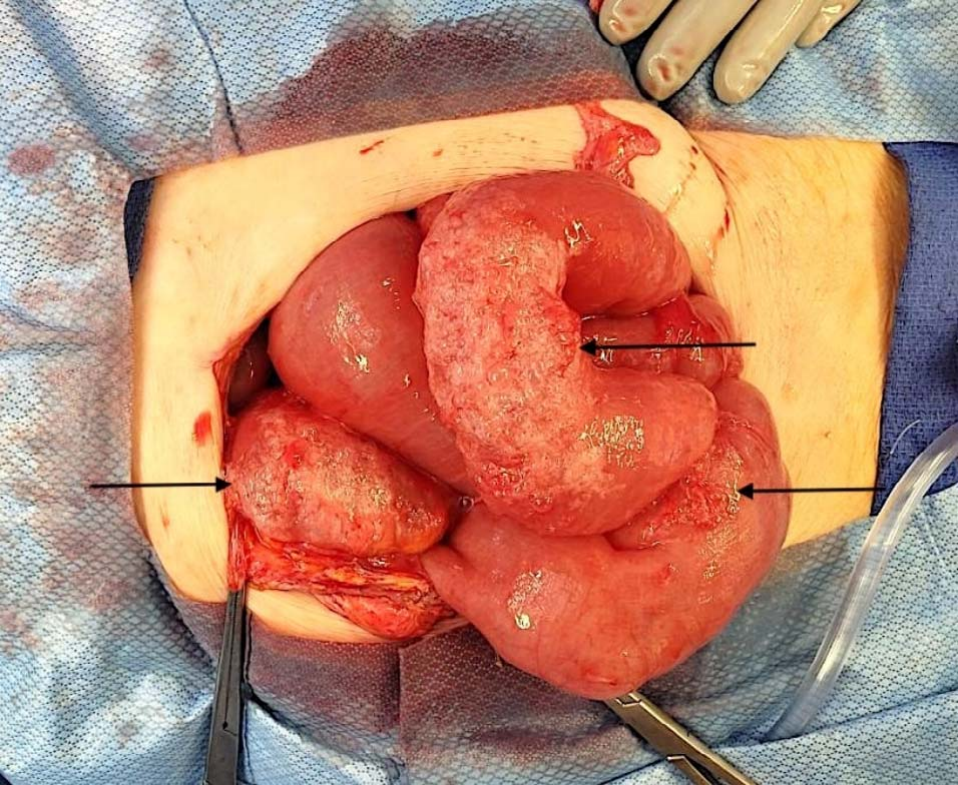
Intraoperative image shows multiple segments of pneumatosis intestinalis (arrows) in the small bowel but no signs of necrosis.

Exploratory laparotomy identified large segments of the small bowel with pneumatosis intestinalis. Despite the presence of pneumatosis intestinalis, the bowel appeared pink and viable with no evidence of perforation, and no free fluid was found in the peritoneal cavity (Figure 3). Bowel resection was not performed, and the patient had an uncomplicated postoperative course. She completed 7 days of empiric antibiotic therapy with metronidazole 500 mg every 8 hours and ciprofloxacin 400 mg every 12 hours, initially given intravenously and then switched to oral once the patient could tolerate diet.

Because of her baseline fragility and hyponatremia, the patient was not discharged until postoperative day 10. At her outpatient postoperative visit 2 weeks after discharge, she was doing well, and no concerns were identified during her physical examination.

## DISCUSSION

DuVernoy first described pneumatosis intestinalis in 1730.^17^ In 1995, the incidence in the general population was reported to be 0.03%^18^; however, advanced imaging techniques have led to greater detection rates, with Morris et al reporting an incidence of 0.37% in 2008.^19^ Pneumatosis intestinalis can occur at any age, with a higher incidence in older patients.^9^ Although the entire gastrointestinal tract can be affected, the small bowel is the most common location (42%), followed by the colon (36%).^20^

Symptoms of pneumatosis intestinalis are not necessarily related to the presence or location of intramural gas in the gastrointestinal tract but rather to the underlying disorder.^2,21^ Moreover, the extent of pneumatosis intestinalis does not correlate with the severity of symptoms or underlying disease.^22^ Presentation is variable, ranging from asymptomatic incidental radiographic findings to a variety of gastrointestinal signs and symptoms such as abdominal distention, pain, nausea, and vomiting.^10,11,21^ In a retrospective analysis of 919 cases of pneumatosis intestinalis, diarrhea, bloody stools, abdominal pain, constipation, weight loss, and tenesmus were the most common related symptoms.^13^ Although pneumatosis intestinalis can arise from benign conditions that are neither obstructive nor ischemic,^9^ life-threatening conditions typically involve bowel ischemia.^6^ Delays in surgical intervention can result in high mortality rates, with reports showing that patients who undergo surgery within <6 hours tend to have a lower mortality rate.^12^ Pneumatosis intestinalis in conjunction with a serum lactate concentration of ≥2.0 mmol/L, hypotension, metabolic acidosis, tachycardia, abdominal tenderness, and a thickened bowel wall has been associated with increased mortality.^12^ Additionally, Hawn et al reported >80% mortality when serum lactate concentration was ≥2.0 mmol/L at the time of pneumatosis intestinalis diagnosis.^23^

While primary pneumatosis intestinalis is rare, a considerable number of diseases and disorders have been implicated as a potential cause.^1,6^ Although the definite pathogenesis of pneumatosis intestinalis remains unclear, mechanical, bacterial, and pulmonary causes have emerged as possible explanations among the various theories of underlying mechanisms behind this pathology.^9,10,11,24^

The mechanical theory proposes that increased intraluminal pressure causes gas migration from the luminal space into the intestinal wall where the gas accumulates to form cysts.^9^ The bacterial hypothesis suggests that air-producing bacteria penetrate the intestinal mucosal barrier, causing gas to become trapped within the submucosa and lymphatic channels.^24^ This theory is supported by the observation that pneumatosis intestinalis has resolved with the use of metronidazole.^15,25^ The pulmonary theory attributes increased intraluminal pressure to the respiratory system.^10,11^ Chronic lung conditions, such as chronic obstructive pulmonary disease, asthma, and interstitial pneumonia can cause alveolar rupture, allowing air to travel through the mediastinum into the perivascular spaces, ultimately reaching the intestinal wall.^10,11,26^

The diagnosis of pneumatosis intestinalis is generally based on findings from plain radiographs or CT scans that demonstrate extraluminal gas in the bowel wall.^2,8^ CT is regarded as the more sensitive diagnostic tool because it can detect important secondary signs that may indicate an underlying cause of pneumatosis intestinalis, including bowel wall thickening, altered mucosal enhancement, abnormally dilated bowel, soft tissue stranding, portal venous gas, and ascites.^27^ Imaging commonly also reveals features suggestive of free intraperitoneal gas.^8^ Importantly, in the setting of pneumatosis intestinalis, pneumoperitoneum alone should not automatically suggest a perforated viscus, as free air can result from the perforation of individual gas-filled cysts.^28^ CT imaging can help identify the distribution of gas and thereby assist in determining the cause of pneumatosis intestinalis.^3^

Useful laboratory tests include a complete blood count with differential, pH, serum bicarbonate, lactic acid, and serum amylase. Knechtle et al reported that the presence of metabolic acidosis, particularly lactic acidosis, and an elevated serum amylase level suggest ischemic bowel.^21^ In the Knechtle et al study correlating laboratory results with ischemic bowel, pH <7.3, serum bicarbonate <20 mmol/L, lactic acid >2 mmol/L, and serum amylase >200 IU/L were predictive of necrotic bowel and were associated with lower survival rates compared to patients with normal values.^21^

Management should be guided by a combination of radiographic findings, physical examination, and a carefully obtained medical history, primarily focusing on determining if the underlying pathology of pneumatosis intestinalis is benign or life-threatening.^9^ Patients with pneumatosis intestinalis on imaging and who present with abdominal pain, tenderness, diarrhea, fever, rectal bleeding, or hypotension are considered to have a severe clinical condition that requires surgery.^21^ However, patients negative for abdominal pain, leukocytosis (white blood cell count <12 mm^3^), sepsis, or acidosis would benefit from medical support and observation.^12^

Therapy is tailored to the suspected etiology. For as many as 50% of patients, treatment is largely supportive and nonspecific.^11^ While infectious causes usually require urgent intervention, pulmonary causes can be managed conservatively.^29,30^ However, because the overall mortality rate in patients with pneumatosis intestinalis ranges from 22% to 50%, nonsurgical management should be approached with caution.^8^

In our case, although the patient was not peritonitic during the physical examination, she continued to have persistent abdominal tenderness in the area of the small bowel pneumatosis intestinalis identified on CT scan. Additionally, despite the absence of acidosis in her laboratory results, the patient arrived with hypotension and leukocytosis, concerns that led us to opt for surgical management to rule out bowel ischemia. The patient had no lung pathology or other conditions that could explain her physical examination findings, laboratory abnormalities, or imaging results.

Conservative management includes observation, hyperbaric oxygen, and antibiotic therapy.^31^ Oxygen treatment is based on creating a high partial pressure of oxygen (PaO_2_) gradient that promotes gas outflow through the cyst wall. The cysts release the gas in them and refill with oxygen, which is subsequently metabolized, leading to resolution.^31^ Antibiotic therapy primarily aims to reduce anaerobic bowel flora to mitigate gas production, and metronidazole is a commonly used agent.^15,25^ Treatment typically continues until clinical and radiologic resolution is achieved.^15^ On the other hand, surgery is the preferred treatment for patients experiencing severe abdominal pain and metabolic derangements such as acidosis, which more likely indicate a state of ischemic or threatened bowel.^1,21,32^

Although our patient ultimately did not benefit from surgical exploration, we believe that when a patient has an unclear clinical presentation, shows some signs of possible bowel ischemia, and does not have an obvious benign cause for pneumatosis intestinalis, a laparotomy may be beneficial.

## CONCLUSION

A thorough evaluation of patients with pneumatosis intestinalis should guide management, with consideration of the patient's history, physical examination, and diagnostic studies, including radiographic patterns. While cases with benign causes can often be treated conservatively, any concerning signs or symptoms that suggest a surgically correctable cause warrant consideration of urgent intervention to reduce the risk of mortality.

## Notes

*This article meets the Accreditation Council for Graduate Medical Education and the American Board of Medical Specialties Maintenance of Certification competencies for Patient Care and Medical Knowledge.*

## References

[1] Braumann C, Menenakos C, Jacobi CA. Pneumatosis intestinalis—a pitfall for surgeons? Scand J Surg. 2005;94(1):47-50. doi: 10.1177/145749690509400112 15865117

[2] St Peter SD, Abbas MA, Kelly KA. The spectrum of pneumatosis intestinalis. Arch Surg. 2003;138(1):68-75. doi: 10.1001/archsurg.138.1.68 12511155

[3] Soyer P, Martin-Grivaud S, Boudiaf M, Linear or bubbly: a pictorial review of CT features of intestinal pneumatosis in adults. Article in French. J Radiol. 2008;89(12):1907-1920. doi: 10.1016/s0221-0363(08)74786-3 19106848

[4] Florin TH, Hills BA. Does counterperfusion supersaturation cause gas cysts in pneumatosis cystoides coli, and can breathing heliox reduce them? Lancet. 1995;345(8959):1220-1222. doi: 10.1016/s0140-6736(95)91996-1 7739311

[5] Lerner HH, Gazin AI. Pneumatosis intestinalis; its roentgenologic diagnosis. Am J Roentgenol Radium Ther. 1946;56:464-469. 21000818

[6] Hwee Hong Lee A, Tellambura S. Pneumatosis intestinalis: not always bowel ischemia. Radiol Case Rep. 2022;17(4):1305-1308. doi: 10.1016/j.radcr.2022.01.062 35242257 PMC8857577

[7] Berritto D, Crincoli R, Iacobellis F, Primary pneumatosis intestinalis of small bowel: a case of a rare disease. Case Rep Surg. 2014;2014:350312. doi: 10.1155/2014/350312 25478280 PMC4248370

[8] Slesser AA, Patel PH, Das SC, Leahy A, Livingstone J, Riaz AA. A rare case of segmental small bowel pneumatosis intestinalis: a case report. Int J Surg Case Rep. 2011;2(7):185-187. doi: 10.1016/j.ijscr.2011.06.003 22096722 PMC3199630

[9] Khalil PN, Huber-Wagner S, Ladurner R, Natural history, clinical pattern, and surgical considerations of pneumatosis intestinalis. Eur J Med Res. 2009;14(6):231-239. doi: 10.1186/2047-783x-14-6-231 19541582 PMC3352014

[10] Gupta AK, Vazquez OA, Lopez-Viego M. Idiopathic pneumatosis of small bowel and bladder. Cureus. 2020;12(5):e8313. doi: 10.7759/cureus.8313 32607296 PMC7320656

[11] Iida A, Naito H, Tsukahara K, Pneumatosis cystoides intestinalis presenting as pneumoperitoneum in a patient with chronic obstructive pulmonary disease: a case report. J Med Case Rep. 2017;11(1):55. doi: 10.1186/s13256-017-1198-2 28241852 PMC5329944

[12] Greenstein AJ, Nguyen SQ, Berlin A, Pneumatosis intestinalis in adults: management, surgical indications, and risk factors for mortality. J Gastrointest Surg. 2007;11(10):1268-1274. doi: 10.1007/s11605-007-0241-9 17687617

[13] Jamart J. Pneumatosis cystoides intestinalis. A statistical study of 919 cases. Acta Hepatogastroenterol (Stuttg). 1979;26(5):419-422. 525221

[14] Lee KS, Hwang S, Hurtado Rúa SM, Janjigian YY, Gollub MJ. Distinguishing benign and life-threatening pneumatosis intestinalis in patients with cancer by CT imaging features. AJR Am J Roentgenol. 2013;200(5):1042-1047. doi: 10.2214/AJR.12.8942 23617487

[15] Bezabih NA, Mehammed AH, Gebresilassie MY, Damtie MY, Midekso HD, Gidna EK. A rare case of extensive pneumatosis cystoides intestinalis with intestinal malrotation: case report. Radiol Case Rep. 2024;19(11):5100-5104. doi: 10.1016/j.radcr.2024.07.145 39253045 PMC11381972

[16] Okuda Y, Mizuno S, Koide T, Suzaki M, Isaji S. Surgical treatment of pneumatosis cystoides intestinalis with pneumoperitoneum secondary. Turk J Gastroenterol. 2018;29(1):131-133. doi: 10.5152/tjg.2018.17519 29391321 PMC6322609

[17] DuVernoy JG. Aer intestinorum tam sub extima quam intima tunica inclusus: observationes anatomicae: comment. Acad Acient Imp Petropol. 1730;5:213-225.

[18] Heng Y, Schuffler MD, Haggitt RC, Rohrmann CA. Pneumatosis intestinalis: a review. Am J Gastroenterol. 1995;90(10):1747-1758. 7572888

[19] Morris MS, Gee AC, Cho SD, Management and outcome of pneumatosis intestinalis. Am J Surg. 2008;195(5):679-683. doi: 10.1016/j.amjsurg.2008.01.011 18424288

[20] Höer J, Truong S, Virnich N, Füzesi L, Schumpelick V. Pneumatosis cystoides intestinalis: confirmation of diagnosis by endoscopic puncture a review of pathogenesis, associated disease and therapy and a new theory of cyst formation. Endoscopy. 1998;30(9):793-799. doi: 10.1055/s-2007-1001424 9932761

[21] Knechtle SJ, Davidoff AM, Rice RP. Pneumatosis intestinalis. Surgical management and clinical outcome. Ann Surg. 1990;212(2):160-165. doi: 10.1097/00000658-199008000-00008 2375647 PMC1358051

[22] Feczko PJ, Mezwa DG, Farah MC, White BD. Clinical significance of pneumatosis of the bowel wall. Radiographics. 1992;12(6):1069-1078. doi: 10.1148/radiographics.12.6.1439012 1439012

[23] Hawn MT, Canon CL, Lockhart ME, Serum lactic acid determines the outcomes of CT diagnosis of pneumatosis of the gastrointestinal tract. Am Surg. 2004;70(1):19-24. 14964540

[24] Keyting WS, McCarver RR, Kovarik JL, Daywitt AL. Pneumatosis intestinalis: a new concept. Radiology. 1961;76:733-741. doi: 10.1148/76.5.733 13752845

[25] Tak PP, Van Duinen CM, Bun P, Pneumatosis cystoides intestinalis in intestinal pseudoobstruction. Resolution after therapy with metronidazole. Dig Dis Sci. 1992;37(6):949-954. doi: 10.1007/BF01300397 1587203

[26] Gillon J, Tadesse K, Logan RF, Holt S, Sircus W. Breath hydrogen in pneumatosis cystoides intestinalis. Gut. 1979;20(11):1008-1011. doi: 10.1136/gut.20.11.1008 527869 PMC1412673

[27] Devgun P, Hassan H. Pneumatosis cystoides intestinalis: a rare benign cause of pneumoperitoneum. Case Rep Radiol. 2013;2013:353245. doi: 10.1155/2013/353245 23984156 PMC3747401

[28] Williams NM, Watkin DF. Spontaneous pneumoperitoneum and other nonsurgical causes of intraperitoneal free gas. Postgrad Med J. 1997;73(863):531-537. doi: 10.1136/pgmj.73.863.531 9373590 PMC2431444

[29] Wayne E, Ough M, Wu A, Management algorithm for pneumatosis intestinalis and portal venous gas: treatment and outcome of 88 consecutive cases. J Gastrointest Surg. 2010;14(3):437-448. doi: 10.1007/s11605-009-1143-9 20077158

[30] Blair HA, Baker R, Albazaz R. Pneumatosis intestinalis an increasingly common radiological finding, benign or life-threatening? A case series. BMJ Case Rep. 2015;2015:bcr2014207234. doi: 10.1136/bcr-2014-207234 PMC4336884 25694632

[31] Calabrese E, Ceponis PJ, Derrick BJ, Moon RE. Successful treatment of pneumatosis intestinalis with associated pneumoperitoneum and ileus with hyperbaric oxygen therapy. BMJ Case Rep. 2017;2017:bcr2017219209. doi: 10.1136/bcr-2017-219209 PMC5747657 28559286

[32] Hwang J, Reddy VS, Sharp KW. Pneumatosis cystoides intestinalis with free intraperitoneal air: a case report. Am Surg. 2003;69(4):346-349. 12716096

